# Risk factors associated with an outbreak of COVID-19 in a meat processing plant in southern Germany, April to June 2020

**DOI:** 10.2807/1560-7917.ES.2022.27.13.2100354

**Published:** 2022-03-31

**Authors:** Iris Finci, Remo Siebenbaum, Josephin Richtzenhain, Angelika Edwards, Carina Rau, Jonas Ehrhardt, Linda Koiou, Brigitte Joggerst, Stefan O Brockmann

**Affiliations:** 1 Baden-Württemberg State Health Office, Stuttgart, Germany; 2 European Programme for Intervention Epidemiology Training (EPIET), ECDC, Stockholm, Sweden; 3 Health Office Enzkreis / Pforzheim, Pforzheim, Germany; 4 Consumer Protection and Veterinary Office Enzkreis, Pforzheim, Germany

**Keywords:** SARS-CoV-2, meat processing plant, risk factors, prevention measures

## Abstract

Meat processing plants have been prominent hotspots for coronavirus disease (COVID-19) outbreaks around the world. We describe infection prevention measures and risk factors for infection spread at a meat processing plant in Germany with a COVID-19 outbreak from April to June 2020. We analysed a cohort of all employees and defined cases as employees with either a PCR or ELISA positive result. Of 1,270 employees, 453 (36%) had evidence of SARS-CoV-2 infection. The highest attack rates were observed in meat processing and slaughtering areas. Multivariable analysis revealed that being a subcontracted employee (adjusted risk ratio (aRR)): 1.43, 95% CI: 1.06–1.96), working in the meat cutting area (aRR: 2.44, 95% CI: 1.45–4.48), working in the slaughtering area (aRR: 2.35, 95% CI: 1.32–4.45) and being a veterinary inspector (aRR: 4.77, 95% CI: 1.16–23.68) increased infection risk. Sharing accommodation or transportation were not identified as risk factors for infection. Our results suggest that workplace was the main risk factor for infection spread. These results highlight the importance of implementing preventive measures targeting meat processing plants. Face masks, distancing, staggering breaks, increased hygiene and regular testing for SARS-CoV2 helped limit this outbreak, as the plant remained open throughout the outbreak.

## Background

Particularly during the first year of the current coronavirus disease (COVID-19) pandemic, clusters have become an important characteristic. Growing evidence exists that large clusters and superspreading events heavily influence the transmission of severe acute respiratory syndrome coronavirus 2 (SARS-CoV-2) [1]. It was estimated that in Hong Kong about 19% of cases seeded 80% of all local transmission [2], and outside of China 10% of cases accounted for 80% of secondary transmissions [3]. Large clusters have been found in connection to indoor and crowded settings such as nursing homes, churches, bars and clubs [4]. Additionally, several countries have reported large clusters of COVID-19 in meat processing plants [5]. Both working conditions and socio-demographic factors might explain why COVID-19 outbreaks are so prominent in meat processing plants [6]. Working conditions do not allow for physical distancing, as employees often stand next to each other on production lines [6]. Moreover, many areas in meat processing plants have low temperatures, which enable the virus to survive [7]. Although the role of air exchange systems in these settings is not clear, air exchange rates and air flow might play an important role in infection spread [8]. Inadequate living conditions in crowded accommodations and shared transportation add to lack of physical distancing [6]. As most employees in meat processing plants are migrants who have potentially precarious employment conditions, reporting symptoms and taking sick leave might be discouraged [9].

These operational practices in meat processing plants can predispose transmission among employees, potentially resulting in large outbreaks that can spill-over to the surrounding population [10]. Therefore, public health measures should address these settings with appropriate prevention measures.

## The outbreak

On 8 April 2020, the local health authority (LHA) was informed of a COVID-19 case who had been hospitalised the previous day. The case investigation revealed that the patient was an employee in a meat processing plant in the beef-cutting department and shared living accommodations with other co-workers. The initial investigation showed that 10 of 11 co-workers who lived in the same building and two additional close contact co-workers were SARS-CoV-2 positive. As a result, exhaustive testing of all employees at the meat processing plant was started on 14 April 2020. On 17 April 2020, upon detection of new cases during the mass PCR testing, the LHA ordered a set of infection prevention measures to be implemented in the meat processing plant.

In this study, we report a large outbreak in a meat processing plant in Germany, describe prevention measures implemented and analyse the risk factors that might have played a role in the infection spread.

## Methods

### Study design

This retrospective cohort study included all employees of the affected meat processing plant.

### Setting

**Figure 1:**
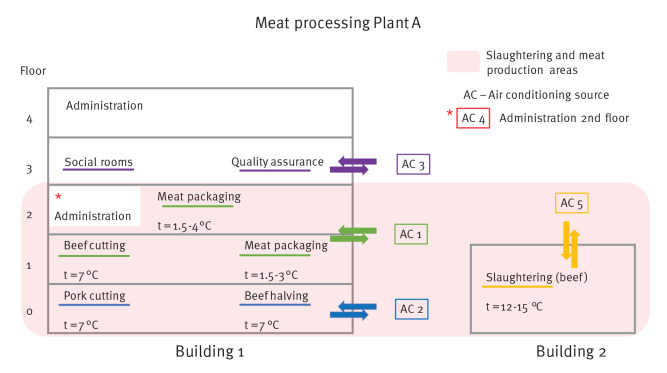
Illustration of the meat processing plant – plan of buildings, floors, air conditioning sources and temperatures

The outbreak occurred in a meat processing plant (including an abattoir) in Germany (plant A). At the time, the plant directly and indirectly employed (i.e., subcontracted) 1,270 people, who lived in three different urban and rural districts. The plant processes both beef and pork and includes two main buildings, a building that includes administration offices, social rooms, quality assurance offices and areas for meat cutting, meat packaging, shipping, sales and transportation (often transportation to off-site locations) (building 1) and a slaughter building (building 2) (Figure 1). The temperature in the meat processing areas (cutting and packaging) was low, between 1.5 and 7 °C. In total, five air conditioning sources were used. Two air conditioning sources were used in the meat processing area, where most the employees worked: air conditioning source 1 was used in packaging and the beef-cutting areas and air conditioning source 2 was used in the pork-cutting and beef halving areas. In the slaughtering department, the temperature was 12–15 °C and air conditioning source 5 was used. Air conditioning sources 3 and 4 were used in two smaller areas in building 1, and source 3 was used in social rooms (changing and break rooms). The employees worked in close proximity (1.5 m and less apart). Generally, there were two working shifts, but the slaughtering area had one shift. Veterinary employees visited slaughtering areas daily, and meat inspection employees visited meat cutting and packaging areas 2–3 times per month. Subcontracted employees took breaks together in a designated breakroom – coffee breaks lasted 15 min and lunch and breakfast breaks 30 min. Veterinary employees sometimes took breaks in the same break rooms. Subcontracted employees lived in large shared accommodations usually with 2–3 people sharing one bedroom and common facilities such as a kitchen, bathroom and toilets, which were shared with additional people. Close contacts were defined as persons having > 15 min face-to-face contact with a confirmed COVID-19 case during infectious period.

Areas in light red are meat production (cutting and packaging) and slaughtering.

### Case definition

Cases were defined as individuals working in the meat processing plant with either a positive PCR test result for SARS-CoV-2 between 7 April and 20 June 2020 or with an ELISA IgG positive result in May or July 2020.

### Laboratory analysis

Nasopharyngeal swab samples were taken from individuals working in the meat processing plant and analysed by an accredited laboratory using RT-qPCR. Serum specimens were tested for IgG antibody reactivity using a SARS-CoV-2 spike protein ELISA (Euroimmun, Lubeck, Germany) as an indicator of prior SARS-CoV-2 infection. For individuals who had a positive PCR test, the time between PCR test and antibody test was longer than 14 days. Antibody tests were performed in May 2020 and July 2020. Individuals who were tested in both periods with discrepant results (borderline and positive) were considered positive.

### Data and analysis

Data on sociodemographic factors and exposures (working area, housing, etc.) of all employees were obtained from the employee roster provided by the employer and were entered into a database. Additionally, in order to examine a possible community transmission following the outbreak, routine surveillance data on COVID-19 collected through the national surveillance system was used to analyse COVID-19 7-day incidence rate in (i) the three urban and rural districts affected by the outbreak, (ii) Baden-Württemberg and (iii) Germany. Using the employee roster, we classified the housing size according to how many workers lived together. To calculate the distance between housing and the meat processing plant, we determined the shortest walking distance using Google Maps. Persons living more than 1 km from the meat processing plant were assumed to have used shared transportation. Exposure to air conditioning sources were based on the work area where employees worked, which was deduced from the air conditioning construction plan of the plant. Legal regulations required serological testing of employees to exempt them from weekly swab testing. The meat processing plant implemented serological testing of its employees. The blood samples were collected at the plant and the meat processing plant operator paid for the serological tests and shared the results of the serological testing with the local health office. Additionally, the majority of blood samples were re-tested at the state health office laboratory.

For statistical analysis, R software was used [11]. Employees’ baseline characteristics were described using median and interquartile range for age and counts and proportions for categorical variables. Univariable and multivariable analysis of exposures was performed using binomial regression. Variables with significant p-value < 0.1 in univariable analysis were progressively added to the multivariable model. In addition, variables that were known to have potential confounding effects were added to the model despite the significance level. When two variables highly correlated with each other, only one was selected for the final model. The following variables were used in the final multivariable model: age, sex, contract, work area, housing size and distance to workplace. Attack rates (AR), risk ratios (RR) and adjusted risk ratios (aRR) with 95% confidence intervals (95% CI) and p values were calculated. Employees with no information on PCR and serological testing were excluded from all the data analysis.

### Ethical statement

Ethical approval was not required since all data were collected in the framework of outbreak investigation. Swabs were taken in the framework of the Infection Protection Act. Voluntary blood samples for serological assay were taken in the framework of occupational medicine in order to comply with the Corona ordinance for slaughterhouses and meat processing [12], which states that meat processing plant employees with evidence of prior SARS-CoV-2 infection were exempted from weekly swab testing. All individuals gave informed consent to have their blood collected.

## Results

### Outbreak description and control measures implementation

**Figure 2:**
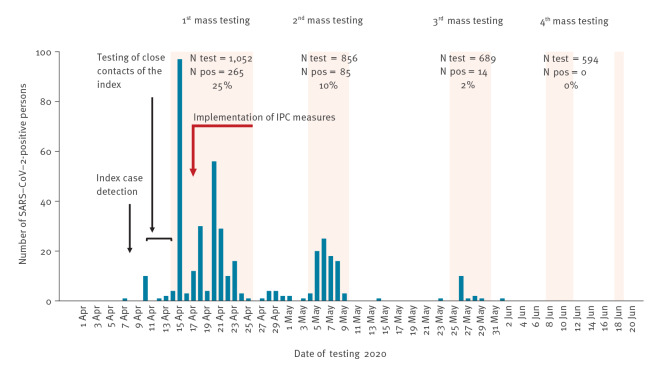
Epidemic curve by PCR testing date of SARS-CoV-2 positive cases in the meat processing plant outbreak

After a positive result of the index and their close contacts (in total 13 positive cases), an outbreak was suspected and four mass testings in plant A were performed. A total of 3,209 PCR tests were conducted, 1,253 of 1,270 employees were tested at least once, and 395 employees had a PCR positive result for SARS-CoV-2. The mass testings had positivity rates of 25% (265/1,052), 10% (85/856) and 2.3% (14/689), and an additional 18 cases were detected between the mass screenings, eight through entry symptom screening. The outbreak finished after no positive test results (0/594) in the last mass testing in June 2020 (Figure 2).

Blue bars represent SARS-CoV-2 positive cases working in meat processing plant. Light orange rectangles represent screenings delegated by the local health authorities in the meat plant. Red arrow depicts timeline when infection prevention and control (IPC) measures were progressively implemented. The epidemic curve shows only cases detected by PCR.

In total, 17 of 395 (4.3%) cases were hospitalised, and none died. Further testing of employees for antibody presence against SARS-CoV-2 discovered an additional 58 employees who had evidence of past SARS-CoV-2 infection, resulting in 453 cases and an overall attack rate of 36%.

Six days after first case detection, mass screening started on 14 April, and on 17 April LHA ordered infection prevention measures to be implemented. All SARS-CoV-2 positive employees were placed in isolation. Isolation was home-based for employees living in private households; if employees lived in shared accommodations, the LHA organised hotels that were used as isolation facilities for asymptomatic and mild cases. Severe cases were hospitalised. The meat processing plant continued to operate with the newly implemented infection control measures described in Table1. All employees with a negative PCR result were considered to have been a close contact of infected individuals and therefore put under ‘working quarantine’. That is, they were allowed to work and to live in their accommodation, but they were not allowed to be in contact with other people outside their work or accommodation and were not allowed to use public transportation.

**Table 1:** Infection prevention and control measures implemented in an outbreak in a meat processing plant, Southern Germany, April to June 2020

Measures	
PCR testing of all personnel	Every 2–3 weeks for the duration of the outbreak, all personnel (except employees with previous positive SARS-CoV-2 PCR result) were repeatedly tested for SARS-CoV-2.
Use of surgical face masks	All employees were provided with surgical face masks that they had to wear at the workplace (not during the breaks).
Physical distancing	Where possible, workstations were separated by 1.5 m, and physical barriers were put between workplaces where separation was not possible.
Cleaning and disinfection	Addition of more hand sanitisers and hand-washing stations, increased frequency of sanitisation of common spaces (canteen and changing rooms), and sanitisation of high-touch areas.
Adjustment of break times and shifts	Separation between two shifts of 1 h. Staggering of break times to avoid crowding. Employees who worked in the same group could go on a break together.
Information and communication in different languages	Team leaders speaking native languages of the employees communicated health promotion and infection prevention messages.
Daily entry symptom screening	All employees were screened before they entered the workplace: temperature checks and questionnaire when symptoms were evident and isolation and testing of symptomatic employees for SARS-CoV-2.
Accommodation inspection	Congregate accommodations were inspected to ensure that hygiene and infection prevention measures were implemented.

### Cohort description

Plant A consisted of 1,270 employees, and 1,258 had evidence of at least one PCR or serological test. In total, 929 (73.8%) were male and the median age was 39 years (range: 17–77). Employees were from Romania (47.1%), Germany (19.7%), Hungary (12.6%) and Poland (8.1%) (complete information on nationality distribution can be found in Supplementary Table S1). More than two thirds of the employees were subcontracted. Overall, 90% of subcontracted employees lived with at least one co-worker, and 74.8% lived with at least 10 co-workers. Most of the subcontracted employees (90%) lived more than 1 km from the meat processing plant. About two thirds of all employees (67.8%) worked in the meat processing area (packaging and cutting), and most of these employees were subcontracted (90.5%).

### Risk factors analysis

The highest attack rates occurred among employees working in the slaughtering and meat production: meat cutting 50.6%, slaughtering 42.6%, meat handling in freezer 35.7%, meat packaging 35.0% and veterinary/meat inspection 38.5% (Table 2). Some of the veterinary employees were infected, but none of the meat inspection employees were infected. The attack rate among subcontracted employees was 43.0%. For employees working in the area with air conditioning sources 1, 2, and 5 attack rates were 44.0%, 36.6%, and 39.7%, respectively.

**Table 2:** Cohort description, attack rates, univariable analysis, and multivariable analysis of risk factors for SARS-CoV-2 infection in outbreak in a meat processing plant, Southern Germany, April to June 2020 (n = 1,258)

Characteristic	N total	% total	N cases	AR (%)	Univariable analysis		Multivariable analysis	
					RR (95% CI)	p value	aRR (95% CI)	p value
Sex								
Male	929	73.8	319	34.0	Ref		Ref	
Female	329	26.2	134	40.4	1.19 (1.01–1.38)	0.034	1.15 (0.98–1.34)	0.084
Age group								
17–29 years	355	28.2	139	38.6	Ref		NA	
30–39 years	285	22.7	88	30.6	0.79 (0.63–0.98)	0.032	NA	
40–49 years	327	26.0	124	37.6	0.97 (0.80–1.17)	0.741	NA	
50–77 years	189	23.1	102	34.9	0.90 (0.73–1.09)	0.286	NA	
Age in years (continuous)					1.00 (0.99–1.00)	0.698	1.01 (1–1.01)	0.103
Type of contract								
Directly contracted	386	30.7	84	21.7	Ref		Ref	
Subcontracted	830	70.0	361	43.0	2.00 (1.64–2.47)	< 0.001	1.43 (1.06–1.96)	0.023
External^a^	42	3.3	8	18.6	0.88 (0.41–1.56)	0.689	0.43 (0.1–1.21)	0.162
Work area								
Administration / office work	84	6.7	13	15.3	Ref		Ref	
Meat packaging	445	35.4	157	35.0	2.28 (1.43–4.05)	0.002	1.61 (0.94–2.97)	0.104
Meat cutting	409	32.5	210	50.6	3.32 (2.10–5.86)	< 0.001	2.44 (1.45–4.48)	0.002
Transport	80	6.4	7	8.8	0.57 (0.22–1.31)	0.197	0.53 (0.21–1.23)	0.155
Slaughtering	61	4.8	26	42.6	2.75 (1.58–5.12)	< 0.001	2.35 (1.32–4.45)	0.005
Cleaning	56	4.5	12	21.4	1.38 (0.67–2.84)	0.368	1.76 (0.82–3.66)	0.128
Shipping	36	2.9	7	19.4	1.26 (0.51–2.81)	0.591	1.18 (0.48–2.67)	0.695
Quality assurance	17	1.4	2	11.8	0.76 (0.13–2.44)	0.700	0.71 (0.12–2.28)	0.631
Meat handling (freezer)	14	1.1	5	35.7	2.31 (0.85–5.15)	0.057	1.66 (0.6–3.86)	0.262
Veterinary/Meat inspection^b^	13	1.0	5	38.5	2.49 (0.93–5.49)	0.036	4.77 (1.16–23.68)	0.036
Other	43	3.4	9	20.0	1.35 (0.6–2.89)	0.440	1.37 (0.62–2.91)	0.412
Air-conditioning source (n = 1,205)								
No source	131	10.4	14	10.6	Ref		NA	
Source 1	641	51.0	285	44.0	4.16 (2.63–7.25)	< 0.001	NA	
Source 2	252	20.0	93	36.6	3.45 (2.14–6.11)	< 0.001	NA	
Source 3	42	3.3	7	16.7	1.56 (0.63–3.49)	0.299	NA	
Source 4	27	2.1	4	14.3	1.39 (0.42–3.53)	0.535	NA	
Source 5	68	5.4	27	39.7	3.72 (2.14–6.85)	< 0.001	NA	
Multiple	44	3.5	9	20.0	1.91 (0.85–4.06)	0.096	NA	
Housing (n employees living together) (n = 1,245)								
1	400	31.8	106	26.3	Ref		Ref	
2–4	145	11.5	44	29.7	1.15 (0.84–1.52)	0.369	0.89 (0.66–1.19)	0.445
5–9	57	4.5	29	50.9	1.92 (1.38–2.55)	< 0.001	1.01 (0.72–1.39)	0.928
10 +	644	51.2	274	42.2	1.61 (1.34–1.94)	< 0.001	0.93 (0.75–1.18)	0.508
Distance to workplace (n = 1,245)								
Less than 1 km	98	7.8	40	40.8	Ref		Ref	
More than 1 km	1147	91.2	413	35.7	0.88 (0.7–1.16)	0.327	1.06 (0.83–1.41)	0.685

AR: attack rate; ARR: adjusted risk ratio; CI: confidence interval; NA: not applicable; Ref: reference; SARS-CoV2: severe acute respiratory syndrome coronavirus 2.

^a^ External employees – employees contracted by another company/organisation whose work is conducted in the plant (e.g., veterinarians, cleaning personnel, etc.

^b^ 2 persons were meat inspection employees; 12 employees had no PCR or serology result (3 female); 10 were subcontracted; 1 directly contracted; 1 external; 6 worked in the meat cutting area; 3 worked in the packaging area; 2 worked in other areas; and 1 worked in administration; all lived more than 1 km from the plant; 5 shared accommodations with  > 10 co-workers; 3 shared accommodations with 2–4 co-workers; and 1 shared accommodations with 1 co-worker.

Case definition: RT-PCR positive result for SARS-CoV-2 or presence for IgG antibody.

Univariable analysis demonstrated that female employees were 1.19 (95% CI: 1.01–1.38) times more likely to get infected. Compared with employees working in administration, employees in slaughtering, meat cutting, meat packaging, meat handling in the freezer and veterinary inspection had higher risk of infection (Table 2). When compared with directly contracted employees, subcontracted employees had a 2.00 (95% CI: 1.64–2.47) times higher risk of infection. Living with more than one co-worker posed higher risk of infection compared with living with no co-workers. However, sub-analysis of subcontracted employees showed no difference in risk between employees living with no co-worker compared with employees living with more than one co-worker (Supplementary Table S2). Lastly, employees working in areas with air conditioning sources 1, 2, and 5 had a 4.16 (95% CI: 2.63–7.25), 3.45 (95% CI: 2.14–6.11), and 3.72 (95% CI: 2.14–6.85) higher risk of infection compared with employees who were generally not exposed to any air conditioning sources (Table 2).

Multivariable analysis revealed that being a subcontracted worker (aRR: 1.43, 95% CI: 1.06–1.96), working in the meat cutting area (aRR: 2.44, 95% CI: 1.45–4.48), working in slaughtering (aRR: 2.35, 95% CI: 1.32–4.45) and being a veterinary inspector (aRR: 4.77, 95% CI: 1.16–23.68) imposed a higher risk of infection. Air conditioning sources were not included in multivariable analysis.

### Symptom entry screening

Data analysis of daily temperature and symptom entry screening implemented after 27 April 2020 identified only 39 persons who either had elevated body temperature (>  37 °C) or symptoms associated with SARS-CoV-2 infection (sore throat, cough, shortness of breath, fatigue, and body and muscle aches). Of these, eight had a SARS-CoV-2 PCR positive test, 26 had negative PCR SARS-CoV-2 test, four were previously positive and had recovered, and one did not have a test result. During the same period, between 27 April and 1 June 2020, PCR mass testing identified 99 employees who were SARS-CoV-2 positive.

### Serological results

**Figure 3:**
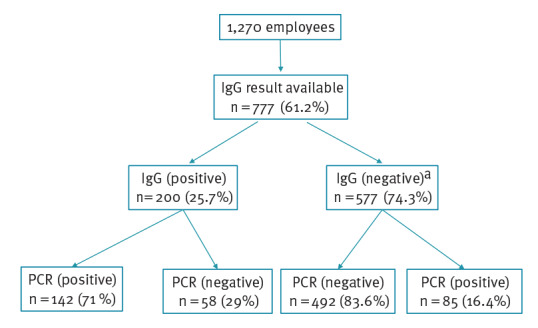
Diagram of serological and PCR testing results of the employees of the meat processing plant in outbreak in a meat processing plant, Southern Germany, April to June 2020

^a^ Negative IgG results include borderline results.

In total, 777 of 1,270 employees were tested for IgG antibodies against SARS-CoV-2 (244 in May and 557 in July 2020; some were tested in May as well as in July). Of these, 200 (25.7%) had IgG positive result (Figure 3) (Supplementary Table S1). Overall, 20.4% of the cases who were identified using antibody testing were not detected using PCR testing (Table 3).

**Table 3:** Distribution of PCR and serological result for COVID-19 cases in outbreak in a meat processing plant, Southern Germany, April to June 2020 (n=285)

	PCR (pos) and IgG (pos)	PCR (pos) and IgG (neg)	PCR (pos) and IgG borderline	PCR (neg) and IgG (pos)	Total
Cases	142 (49.8%)	67 (23.5%)	18 (6.3%)	58 (20.4%)	285

Neg: negative; pos: positive.

Case definition: PCR or IgG positive result; in the table above only cases with both PCR and serological results available are listed

### Spill over to surrounding population

Analysis of the incidence of COVID-19 cases in three urban and rural districts affected by the outbreak, the state of Baden-Württemberg, and all of Germany did not show differences in incidence after removing all cases detected in the meat processing plant (Supplementary Figure S1, comparison of 7-day incidence of SARS-CoV-2 at the levels of affected districts, state and Germany).

## Discussion

In this study, we described the unfolding of a large COVID-19 outbreak in a meat processing plant. This event required extensive implementation of infection prevention measures and outbreak management. Furthermore, we identified potential risk factors for SARS-CoV-2 infection spread in the meat processing plant.

It is estimated that in Germany two thirds of meat processing plant employees are subcontracted and mainly originate from outside Germany [13]. In our study, subcontracted employees also constituted two thirds of all employees and were the most affected by COVID-19, with 43% higher risk of getting infected compared with directly contracted employees.

Working in meat cutting and slaughtering areas imposed a higher risk of infection not only in the subcontracted workers but also in the veterinarians, who monitored the slaughtering areas on a daily basis. In contrast, meat inspection employees, who visited meat cutting areas infrequently (2–3 times per month), did not get infected, possibly due to less frequent exposure. These results suggest that working conditions are an important factor in the spread of infection. Employees work close to each other (less than 1.5 m apart). The work tasks require hard physical work, leading to heavy breathing, which could have resulted in a lack of mask wearing adherence [6]. Due to the noise levels of machinery, employees have to speak loudly, which can contribute to transmission via aerosol emission [14]. Furthermore, meat cutting was situated in big halls where temperatures are very low, between 4 and 7 °C, and the relative humidity is high. SARS-CoV-2 has high stability at lower temperatures [7] and survives longer on metallic surfaces such as the ones used in the plant [15]. Air conditioning sources overlapped with working areas, so it is hard to determine whether the air exchange system itself was a risk factor in this setting. However, low air exchange rate and cooled air recirculation potentially promote the spread of the virus [8]. In another meat processing plant outbreak, an index case transmitted SARS‐CoV‐2 to co‐workers over distances of more than 8 m in a setting where the air was constantly recirculated and cooled [16].

Subcontracted employees may have had exposures outside the production areas as they were taking breaks together in a dedicated break/lunch room. This room was mostly used by them although sometimes the veterinary employees used the same room. Exposure in the break rooms could have been an important risk factor for virus transmission, as employees were physically close and socialised during the meals without masks. Similarly, in an outbreak in a hospital setting, the virus transmission possibly happened in the breakrooms, as co-workers were not perceived as a threat for SARS-CoV-2 transmission so masks were not worn [17].

In addition to the workplace related risk of infection, subcontracted employees may have had further exposures outside the workplace. They often lived together in shared accommodations and shared transportation to the workplace. However, sub-analysis of subcontracted employees and multivariable analysis both showed the risk of being infected for employees living in shared accommodations with 10 or more co-workers was not higher compared with employees not living with co-workers. Moreover, we assumed that subcontracted employees living less than 1 km from the plant did not share transportation, and they had the same risk of getting infected as employees who lived further away and reported using shared transportation to work. These two results suggest that shared accommodation and transportation were not the main risk factors for SARS-CoV-2 infection in this particular setting, a finding that is in accordance with similar outbreaks in Germany and the US [8,18].

To prevent outbreaks of COVID-19 in meat and other food processing plants, targeted infection prevention measures, such as the ones taken for this outbreak, should be implemented. In the workplace, consistent and correct use of masks should be encouraged and monitored. A minimum distance of 1.5 m between workstations should be ensured and when this is not possible, physical barriers should be installed. The ventilation should be improved so that the recirculated air is avoided or reduced, and the air exchange rate (outdoor air change rate) should be increased [19].

Another hypothesis why meat processing plants are disproportionately affected is hesitancy to report symptoms due to precarious working conditions of many migrant employees. However, the results of daily entry symptoms and temperature screening demonstrated that from thousands of entry screenings, only eight COVID-19 cases were detected. As two thirds of employees were younger than 40 years, they might have been asymptomatic or with mild symptoms and therefore were not detected through symptom screening. Based on our data as well as a study of a US Navy ship crew [20], symptom-based screening might not be an efficient way to detect SARS-CoV-2 positive cases in such settings. A more sensitive method is the regular swab testing of employees as implemented in Baden-Württemberg together with mandatory PCR test results for new employees [12].

The non-production settings should be specifically targeted as employees tend to crowd in social rooms such as changing rooms and breakrooms and lunchrooms. Separation of shifts and working teams will help avoid unnecessary contacts between employees. In the breakrooms and lunchrooms, the maximum number of persons should be limited, and tables should be separated as employees do not wear masks while eating or smoking. If possible, outdoor break areas should be used.

During shared transportation, employees should wear masks to avoid virus transmission. Basic hygiene measures should be ensured in shared accommodations. In similar settings with high proportion of migrant employees, communication and health messaging dissemination should be done in the native languages and adjusted to cultural background. This communication should include advice on how to avoid virus spread in the accommodations. Such a tailored approach should help increase awareness, acceptance and compliance with infection prevention measures [21].

Large outbreaks may spill over into surrounding communities causing an increase in the local case numbers, which can lead to a lockdown in the affected area [22]. The analysis of the weekly incidence in the three affected urban and rural districts showed that there was no obvious increase in incidence after the outbreak in the meat processing plant (Supplementary Figure S1). One possible explanation could be that subcontracted employees do not interact much with the local community due to language and social barriers. Additionally, cases were detected and isolated quickly and the working quarantine was implemented early, which also halted possible spillovers to local communities.

Additional serological investigation for a subcohort of employees showed that about 20% of all cases were detected only by serology (i.e., not PCR). They were potentially infected before the outbreak was recognised. Overall, 36% of all employees had either a PCR or antibody positive test. Hence, we think that having such large proportion of employees infected may have contributed to limiting the infection spread. However, completely halting the outbreak required the implementation of strict preventive measures.

The strength of this study lies within the cohort design together with the availability of detailed data on working conditions, housing, etc. There are also several limitations in this study. As date of symptom onset was generally not available due to most cases being asymptomatic, identifying patient zero and how the infection was introduced is difficult. Overall, 29% of persons did not have evidence of immune response despite having a PCR result confirming a SARS-CoV-2 infection. The distribution of PCR positive results over multiple dates for these persons reduces the possibility of false positive PCR results due to contamination. The discrepancy might be explained by the combination of sensitivity of ELISA together with lower percent of IgG response and possible waning of IgG antibodies after 8 weeks in asymptomatic persons [23]. Shared transportation to the workplace was based on distance from housing to the meat processing plant and therefore is only an approximation. However, no other data on this factor were available. Whole genome sequencing was not performed, so we do not know if this was a single introduction and subsequently a superspreading event as shown in another meat processing plant [8] or if multiple independent introductions happened simultaneously as was detected in several retirement homes in the UK [24]. Because of community transmission, some of the cases might have been infected outside of the workplace. No environmental samples from the meat processing plant were available. Nonetheless, in future outbreaks in meat processing plants, taking samples from air conditioning sources would be helpful as it would allow further understanding of the involvement of air conditioning and air exchange systems in viral particle spread.

## Conclusions

We described a successful control of a large outbreak of COVID-19 in a meat processing plant that may support prevention of similar outbreaks. Our results suggest that workplace is a factor involved in infection spread; therefore, preventive measures targeting the workplace should be implemented to prevent infection spread. Outbreaks that occurred in slaughterhouses and meat processing plants led to a law in Germany requiring the monitoring of workers’ protection (Occupational Safety and Health Control Act), which came into force on 1 January 2021 [25]. The law reform bans outsourcing slaughtering and cutting work to subcontracted workers (i.e., these workers are now hired directly by the meat processing plants). In addition, the law strengthens the enforcement of occupational health protection standards as well as implements clear protection standards for shared accommodations of workers in meat processing plants.

## Supplementary Data

165000 Supplementary Material
