# Supplementary material for: Risk factors associated with an outbreak of COVID-19 in a meat processing plant in southern Germany, April to June 2020
Source: Euro Surveill. 2022 Mar 31;27(13):2100354. doi: 10.2807/1560-7917.ES.2022.27.13.2100354 (PMC8973015; doi:10.2807/1560-7917.ES.2022.27.13.2100354)
Supplement: Supplementary Material [file 21-00354_FINCI_Supplementary_material.pdf]

Supplementary material

This supplementary material is hosted by *Eurosurveillance* as supporting information alongside the article 'Risk factors associated with an outbreak of COVID-19 in a meat processing plant in southern Germany, April to June 2020', on behalf of the authors, who remain responsible for the accuracy and appropriateness of the content. The same standards for ethics, copyright, attributions and permissions as for the article apply. Supplements are not edited by *Eurosurveillance* and the journal is not responsible for the maintenance of any links or email addresses provided therein.

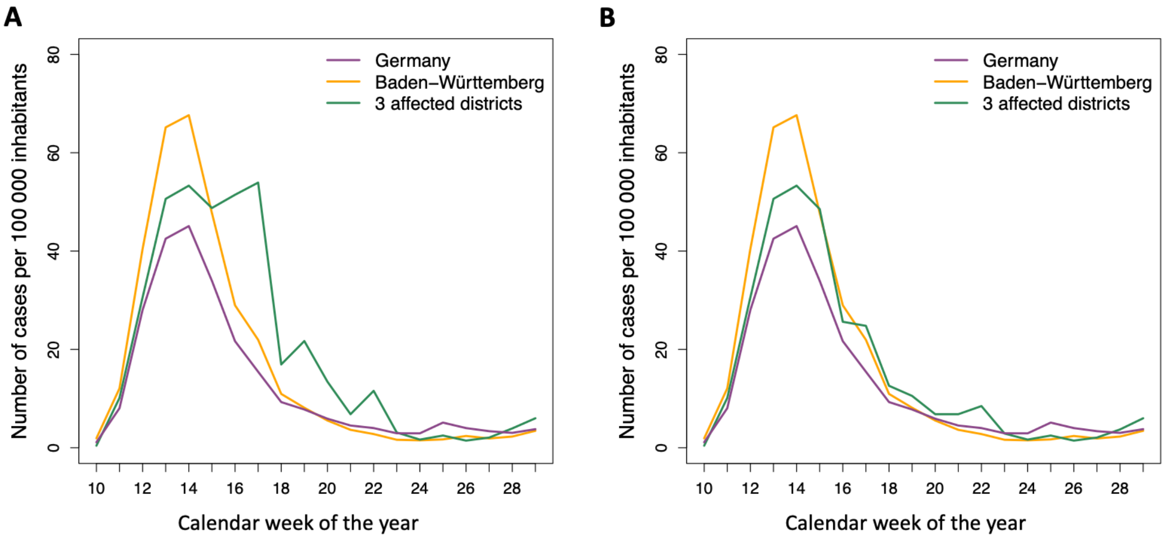

Figure S1. Seven-day incidence of SARS-CoV-2 positive cases per 100 000 inhabitants in three affected districts, Baden-Württemberg and Germany, calendar week 10-29, 2020, A – includes all cases from the meat processing plant outbreak; B –cases from the meat processing plant outbreak are excluded from data of Baden-Württemberg and three affected districts

Table S1. Attack rates, univariable and multivariable analysis of risk factors for SARS-CoV-2 infection. Case definition: RT-PCR positive result for SARS-CoV-2 or presence for IgG antibody

Factor		N total	% total	N case	AR (%)	RR (95% CI)	pval
Nationality N=1,220	German	248	20.3	52	21.0	ref	
	Romanian	592	48.5	270	45.0	3.16 (2.25-4.5)	<0.001***
	Hungarian	159	12.9	96	39.4	2.47 (1.59-3.86)	<0.001***
	Polish	102	8.3	68	33.0	1.88 (1.12-3.14)	0.015*
	Croatian	29	2.4	3	10.3	0.43 (0.1-1.3)	0.186
	Bulgarian	21	1.7	4	19.0	0.89 (0.25-2.52)	0.835
	Iraqi	18	1.5	4	21.1	1.08 (0.3-3.15)	0.900
	other	107	8.5	14	27.5	1.43 (0.7-2.79)	0.311
Housing (N subcontracted employees living together)	1	83	10.0	38	45.2	ref	
	2-4	71	8.6	25	33.8	0.64 (0.33-1.23)	0.185
	5-9	55	6.7	29	52.7	1.32 (0.67-2.63)	0.425
	10+	618	74.8	269	43.2	0.91 (0.58-1.45)	0.697

N=827							
Distance to work – transport use (sub-contracted employees) N=827	Less than 1 km	78	9.4	37	47.4	ref	
	More than 1 km	749	90.6	324	42.7	0.84 (0.53-1.35)	0.479

Table S2. Comparison of PCR results and serological results for a subcohort of 777 meat processing plant employees

	**PCR positive**	**PCR not positive**	**Total**
**IgG positive**	142	58	**200**
**IgG negative**	67	482	**549**
**IgG borderline**	18	10	**28**
**Total**	**227**	**550**	777
